# Contraction and Hydroscopic Expansion Stress of Dental Ion-Releasing Polymeric Materials

**DOI:** 10.3390/polym10101093

**Published:** 2018-10-02

**Authors:** Krzysztof Sokolowski, Agata Szczesio-Wlodarczyk, Kinga Bociong, Michal Krasowski, Magdalena Fronczek-Wojciechowska, Monika Domarecka, Jerzy Sokolowski, Monika Lukomska-Szymanska

**Affiliations:** 1 Department of Restorative Dentistry, 251 Pomorska St., 92-213 Lodz, Poland; krzysztof.sokolowski@umed.lodz.pl; 2 University Laboratory of Materials Research, Medical University of Lodz, 251 Pomorska St., 92-213 Lodz, Poland; agata.szczesio@umed.lodz.pl (A.S.-W.); kinga.bociong@umed.lodz.pl (K.B.); michal.krasowski@umed.lodz.pl (M.K.); 3 “DynamoLab” Academic Laboratory of Movement and Human Physical Performance, Medical University of Lodz, 251 Pomorska St., 92-216 Lodz, Poland; magdalena.fronczek-wojciechowska@umed.lodz.pl; 4 Department of General Dentistry, Medical University of Lodz, 251 Pomorska St., 92-213 Lodz, Poland; monika.domarecka@umed.lodz.pl (M.D.); jerzy.sokolowski@umed.lodz.pl (J.S.)

**Keywords:** ion-releasing materials, shrinkage stress, water sorption, hydroscopic expansion, photoelastic investigation

## Abstract

Ion-releasing polymeric restorative materials seem to be promising solutions, due to their possible anticaries effect. However, acid functional groups (monomers) and glass filler increase hydrophilicity and, supposedly, water sorption. The purpose of the study was to evaluate the influence of water sorption of polymeric materials on the stress state at the restoration-tooth interface. Beautifil Bulk Fill Flow, Beautifil Flow Plus F00, Beautifil Flow F02, Dyract eXtra, Compoglass Flow, Ionosit, Glasiosite, TwinkiStar, Ionolux and Fuji II LC were used for the study. The stress state was measured using photoelastic analysis after: 0.5, 24, 72, 96, 168, 240, 336, 504, 672, 1344 and 2016 h. Moreover, water sorption, solubility and absorption dynamic were assessed. The water sorption, solubility and absorption dynamic of ion-releasing restorative materials are material dependent properties. The overall results indicated that the tested restorative materials showed significant stress decrease. The total reduction in contraction stress and water expansion stress was not observed for materials with low value of water sorption (Beautifil Bulk Fill, Dyract eXtra, Glasionosit and Twinky Star). The photoelastic method turned out to be inadequate to evaluate stress changes of resin modified glass-ionomer cement (RMGI, Fuji II LC and Ionolux).

## 1. Introduction

Development in dentistry is driven by the desire to create a restorative material exhibiting high aesthetics, biocompatibility, durability and permanent adhesion to tooth structure [1]. Many products have been developed aiming at meeting these expectations. Dentists perceive fluoride-releasing restorative materials as being very attractive due to their advertised multifunction performance. Besides esthetic and restoring function, these materials could prevent or arrest the progression of caries lesions [2]. The glass-ionomer cements (GICs) release fluoride ions and are biocompatible, however, they exhibit inherent drawbacks such as low compressive strength, early moisture sensitivity and inadequate esthetics [3,4]. Resin modified glass-ionomer cement (RMGIC) was designed to combine chemistry of GIC with dental resins. This material shows favorable properties similar to composites while maintaining the features of the conventional GIC [5]. Acid/base reaction predominates in overall curing process of RMGIs. An additional curing process is the photo-polymerization of methacrylate monomers that are attached to molecules of the acidic liquid component [6]. Compomers provide benefits of composites (the “comp” in their name) and glass-ionomers (“omer”). These materials contain dimethacrylate monomers with acid functional group and partially salinized ion-leachable glass. These components may participate in an acid/base glass-ionomer reaction following polymerization of the resin molecule. However, this process probably does not influence the overall properties of these materials. Considering the internal structure, these materials are classified as a new type of composites [7,8]. Giomers were developed to combine the advantages of composites (good mechanical and esthetic properties) and GICs (anticariogenic activity). Being resin-based materials, they contain surface reaction-type pre-reacted glass-ionomer (S-PRG) particles. These particles are made of fluorosilicate glass that react with polyacrylic acid prior to being incorporated into the resin [9].

The polymerization shrinkage is an integral feature of all currently available resin-based/polymer materials [10,11]. Previous studies showed the reduction in the initial stress state (generated by photo-polymerization) in composite materials by hygroscopic expansion [12,13]. The value of contraction stress and the magnitude of stress reduction were proven to be material-dependent [12,14]. Moreover, acid functional groups in monomers and glass filler increased hydrophilicity and supposedly water sorption of materials that combined chemistry of GIC and resin composites. As a result, the water sorption and the change of contraction stress generated during curing might be significantly modified. The in-depth analysis of the change in stress state for compomers and giomers after ageing in water is still missing. The purpose of the study was to evaluate the influence of water sorption of selected ion-releasing polymeric restorative materials on the stress state at the restoration-tooth interface.

## 2. Materials and Methods 

The composition of investigated materials and bonding systems is presented in Table 1 and Table 2. To ensure consistent irradiance values, the light curing units (Mini L.E.D, Satelec, France) were calibrated with a radiometer system (Digital Light Meter 200 from Rolence Enterprice Inc., Taoyuan, Taiwan). Increments of 2 mm in thickness were polymerized. Materials were tested after a selected period of time (30 min, 24, 72, 96, 168, 240, 336, 504, 672, 1344 and 2016 h).

### 2.1. Water Absorption Dynamic Study

The detailed procedure for determination of absorption dynamic has also been described in our earlier works [12,14]. In order to characterize absorbency dynamic the five cylindrical samples—15 mm in diameter and 1 mm in width—were prepared according to ISO 4049 [15]. Curing time was consistent with the manufacturers’ instructions (Table 1 and Table 2). Light curing units exhibited an output irradiance of 1250 mW/cm^2^, as stated by the manufacturer. 

Absorption is a physical or chemical phenomenon or a process in which atoms, molecules or ions enter some bulk phase—gas, liquid or solid material. This is a different process from adsorption, since molecules undergoing absorption are taken up by the volume, not by the surface (as in the case for adsorption). A more general term is sorption, which covers absorption, adsorption, and ion exchange. The main phenomenon observed in the present study is the absorption. 

The samples were weighted (RADWAG AS 160/C/2, Poland) immediately after preparation and then daily for 30 days, and after 1344 h (56 days) and 2016 h (84 days).

Water absorption was calculated according to following equations:

(1) $$\;A=\frac{m_{i}-m_{0}}{m_{0}}\cdot 100\%\;\;$$

where: *A*—water absorption, *m*_0_—the mass of the sample in dry condition [g] and *m_i_*—mass of the sample after storage in water for a specified (*i*) period of time.

### 2.2. Water Sorption and Solubility

Water sorption and solubility was investigated according to ISO 4049 [15]. Five samples were prepared for each dental material. The samples were prepared using the silicone mold (15 mm in diameter, 1 mm in width). Tested materials were cured in nine zones partially overlapping. Exposure time was consistent with the manufacturer instructions (Table 2). Direct contact of optical fiber with the sample surface was ensured. Specimens were placed in a vacuum desiccator (Duran^®^, Mainz, Germany) at a temperature of 37 ± 1 °C for 23 h, transferred to a second desiccator at the temperature of 25 ± 1 °C for 1 h, and then weighed in the balance. This cycle was repeated until a constant mass was obtained (*m*_1_). Upon stabilization, specimens were immersed in distilled water at a temperature of 37 ± 1 °C for 7 days. Specimens were removed, gently dried with absorbent paper, and weighed again to obtain *m*_2_. Using the same protocol as for m_1_, specimens were then reconditioned in the desiccators until a constant mass was obtained (*m*_3_). 

Water sorption and solubility were calculated according to following equations:

(2) $$\;W_{sp}=\frac{m_{2}-m_{3}}{V}\;\;$$

(3) $$\;W_{sl}=\frac{m_{1}-m_{3}}{V}\;\;$$

where: *W_sp_*—water sorption, *W_sl_*—water solubility, *m*_1_—initial constant mass [μg], *m*_2_—mass after 7 days of water immersion [μg], *m*_3_—final constant mass [μg] and *V*—specimen volume [mm^3^].

### 2.3. Photoelastic Study

The modified photoelastic analysis method enables analysis of the relationship between water sorption and the change of stress state (contraction or expansion) of materials with resin matrix. This test was extensively described [12,14]. Photoelastically sensitive plates of epoxy resin (Epidian 53, Organika-Sarzyna SA, Nowa Sarzyna, Poland) were used in this study. Calibrated orifices of 3 mm in diameter and of 4 mm in depth were prepared in resin plates in order to mimic an average tooth cavity. The generated strains in the plates were visualized in circular transmission polariscope FL200 (Gunt, Germany). Next, photoelastic strain calculations were based on the Timoshenko equation [16]. Three samples were prepared for each material. Curing time was consistent with the manufacturers’ instructions (Table 1 and Table 2). 

## 3. Results

### 3.1. Absorption Dynamic Study

**Figure 1:**
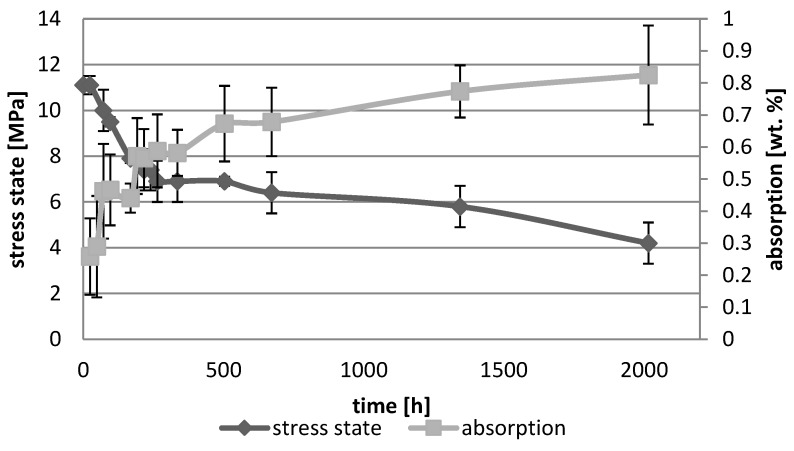
The relationship of absorption and stress state during water ageing (2016 h) of Beautifil Bulk Flow.

**Figure 2:**
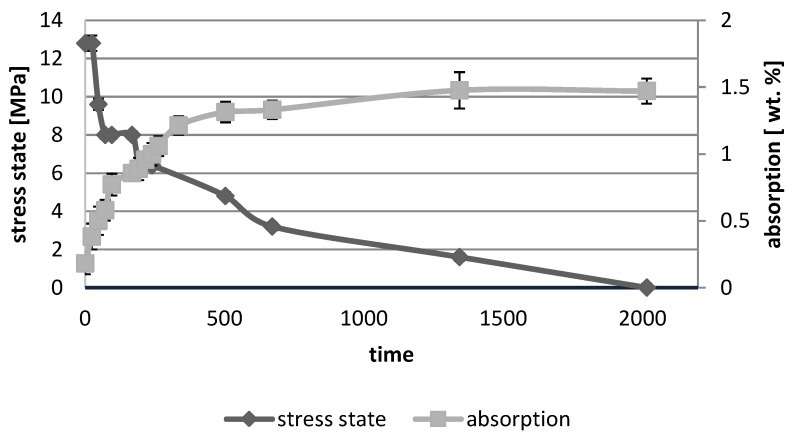
The relationship of absorption and stress state during water ageing (2016 h) of Beautifil Flow Plus F00.

**Figure 3:**
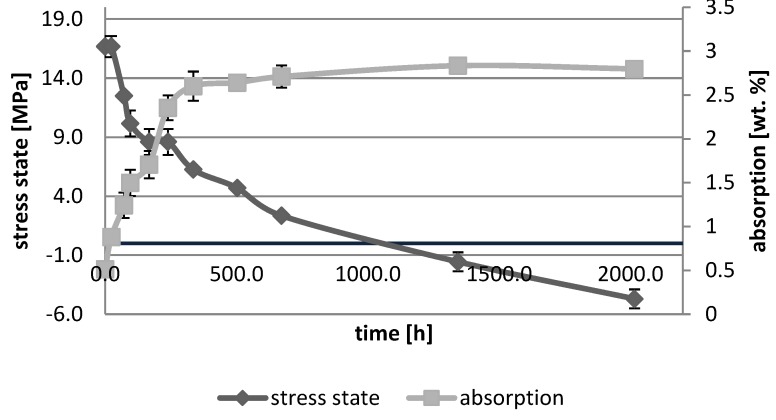
The relationship of absorption and stress state during water ageing (2016 h) of Beautifil Flow F02.

**Figure 4:**
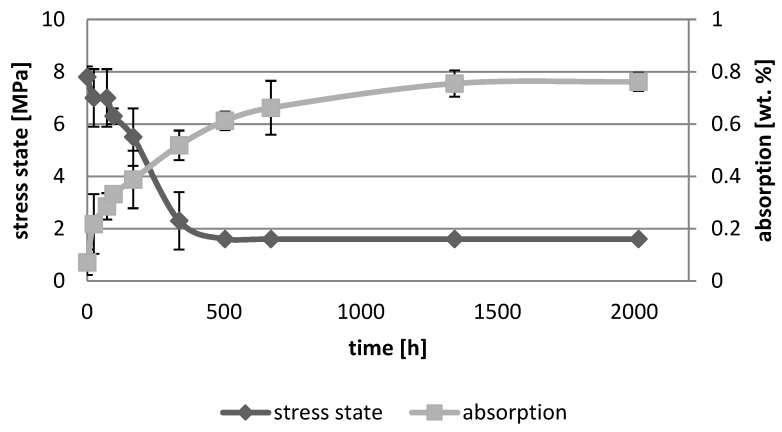
The relationship of absorption and stress state during water ageing (2016 h) of Dyract eXtra.

**Figure 5:**
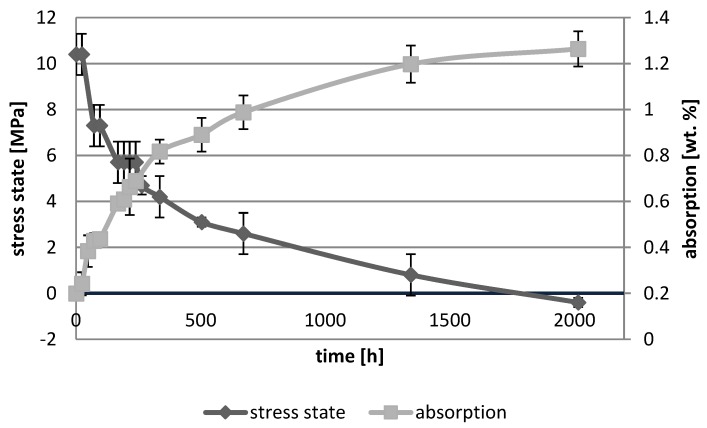
The relationship of absorption and stress state during water ageing (2016 h) of Compoglass Flow.

**Figure 6:**
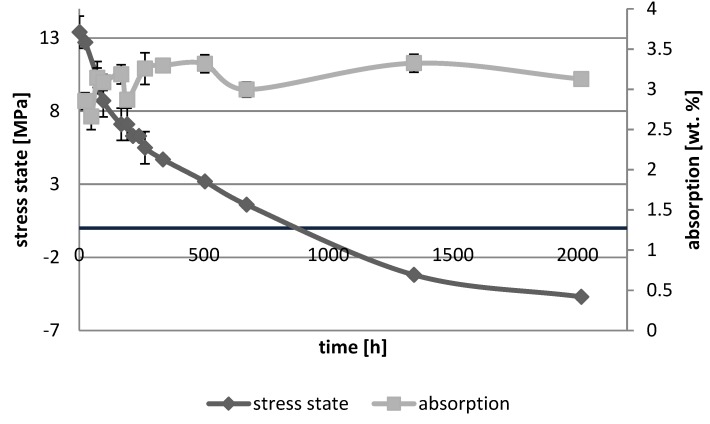
The relationship of absorption and stress state during water ageing (2016 h) of Ionosit.

**Figure 7:**
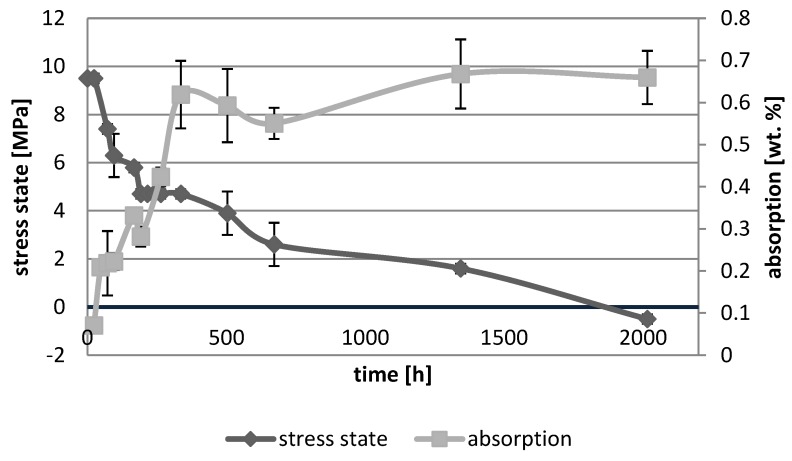
The relationship of absorption and stress state during water ageing (2016 h) of Glasiosite.

**Figure 8:**
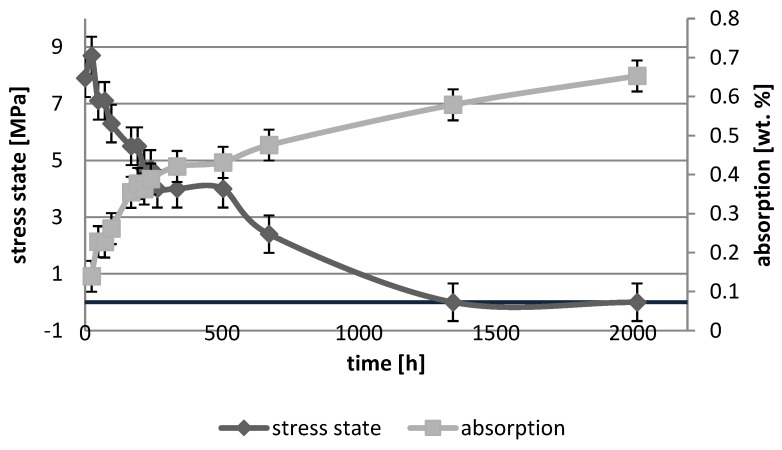
The relationship of absorption and stress state during water ageing (2016 h) of Twinky Star.

Figure 1, Figure 2, Figure 3, Figure 4, Figure 5, Figure 6, Figure 7 and Figure 8 presented mean values and standard deviations of water absorbency and stress state. The water immersion resulted in an increase in weight of all tested materials. The water sorption (weight %) increased for Beautifil Flow F02 and Ionosit up to 3 wt % (Figure 3 and Figure 6). Twinky Star showed the lowest value of absorption after 2016 h (84 days) (Figure 8). 

### 3.2. Water Sorption and Solubility

Mean values of water sorption and solubility were presented in Table 3. Ionosit and Beautifil Flow F02 exhibited the highest, while Beautifil Bulk Fill Flow the lowest values of water sorption. The water sorption of the selected RMGI amounted to more than 115 µg/mm^3^.

### 3.3. Photoelastic Study

**Figure 9:**
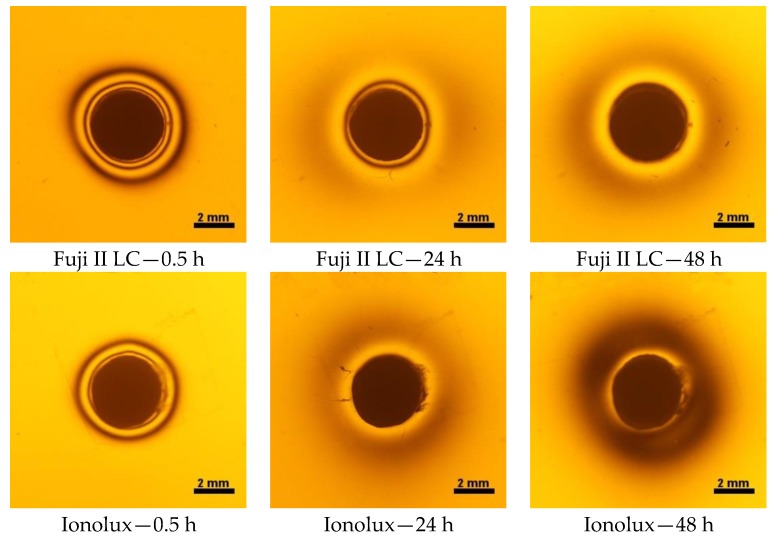
Isochromes in epoxy plate around Fuji II LC and Ionolux 0.5 h and after 24 and 48 h of water storage.

The polymerization shrinkage stress for RMGI (Fuji II LC and Ionolux) was observed after 30 min. Based on photoelastic images, additional isochromes around restoration appeared after 24 h (Figure 9). They occurred during the transition from the contraction stress to the expansion stress (resulting from the material sorption). However, isochromes generated by contraction were still visible and the analysis of the state stress changes using this research method was impossible. It might be assumed that the material undergone remodeling process during the acid-base reaction [6] after 24 h. 

In contrast, the changes in the stress state resulting from the water sorption in compomers and giomers was feasible. The results were presented in Section 3.1, Section 3.2 and Section 3.3.

All ion-releasing restorative materials shrank during hardening process and exhibited the associated contraction stress. The significant decrease in the stress state was observed due to hygroscopic expansion of materials (Figure 1, Figure 2, Figure 3, Figure 4, Figure 5, Figure 6, Figure 7 and Figure 8). The absolute values of stress changes amounted from 6.2 up to 21.4 MPa (Table 3). Upon water aging, most of the tested materials showed total decrease in stress state (100%) and four materials showed additional stress characterized by the opposite direction of forces (expansion stress).

## 4. Discussion

Ion-releasing restorative materials are a substantial part of modern restorative dentistry. Released fluoride may be incorporated into the surrounding enamel or dentin with anti-cariogenic effect. These materials complement their fluoride reservoir from the oral cavity by building in fluoride delivered via rinses, toothpastes or topical application of fluoride preparations. Thus, the filling is a local fluoride storage that is used when the decrease in pH level occurs [17,18]. However, these materials exhibit significant water sorption, which may affect the formation of expansion stress endangering the integrity of tooth structure. Therefore, the profound knowledge of the rate and extent of water sorption of these materials is of paramount importance.

In the present study, the overall results indicated that the ion-releasing restorative materials showed greater stress decrease as compared with resin composites [12]. It might be associated with the process of ion release due to various types of fluoride glasses used as fillers. However, no significant difference between giomers and compomers was found. Discrepancies occurred between individual materials and depended on the material composition. Giomers consisted mainly of bis-GMA/TEGDMA resin matrix and multifunctional glass filler based on alumino-fluoro-borosilicate glass (Table 1). The differences resulted primarily from the filler amount in the materials. It is worth emphasizing, that the increase in filler content caused the decrease in sorption and solubility of materials. These phenomena might be explained by the reduction of free volume in polymer matrix [19,20]. Beautifil Bulk Fill Flow showed the lowest sorption value (13.6 µg/mm^3^) due to the addition of UDMA and bis-MEPEPP resins. Both resins exhibited lower water sorption than bis-GMA and TEGDMA [21,22]. The strong relationship between the value of water sorption and the change in the stress state of giomers was observed; the higher the water sorption in the giomer, the greater the relaxation effect of stress (resulting from the plasticization effect and hydroscopic expansion). Beautifil Flow F02 showed high values of sorption and expansion stress after 1344 h of water ageing. The water sorption of Beautifil Flow Plus F00 amounted up to 26.4 μg/mm^3^. 

The water absorption of any material was controlled by a variety of mechanisms, but the osmotic effect led to an increased radial pressure. Since the Beautifil Flow F02 (giomer) demonstrated significant swelling along with the greatest radial pressure, it might be assumed that this material - more likely than any other studied material - was able to generate a significant osmotic effect. The main difference in microstructure between the giomers and compomers was the presence of pre-reacted glass-polyacid zones in the giomer structure (as a part of the filler) [23]. It seemed likely that these zones were responsible for generating the osmotic effect which led to swelling and pressure generation. Similar zones could be potentially formed at the surface of compomer glass particles following the delayed acid-base reaction which occurred due to water absorption. The latter reaction (in compomers) was significantly limited in extent compared with the almost total consumption of glass (in giomers). However, Ionosit (compomer) produced a significant radial pressure after 56 days of water storage. These results corresponded with the study of McCabe at al., showing compomer (F2000) exhibiting high displacement force and radial pressure due to water storage for 1 month [24]. 

Most compomers and giomers showed an extended process of water sorption. UDMA and a high degree of filler content were used to limit water sorption [19]. The high water sorption could be explained by the occurrence of later acid-base reaction in giomers. The setting process of compomers, in contrast to GIC, was based on photopolymerization and chemical reactions between glass particle fillers, while the reaction of monomers with acid functional group was only additional [25]. Compomers contained hydrophilic resins that were copolymerized with the more hydrophobic urethane dimethacrylate or bis-GMA [23]. The initial water uptake of these materials was lower than that of RMGICs, however, it was considerably higher than that of resin composites [12,14]. While compomers were initially anhydrous when polymerized, the dynamic synergistic interaction between the ion-leachable fillers and the acidic resin phase (resin matrix) was initiated upon water sorption [26]. The acid-base reaction in compomers invariably led to the formation of hydrogel layers around the glass fillers and an irreversible increase in the amount of ‘firmly-bound’ water [26]. The additional phase of resin matrix expansion to accommodate the swollen hydrogel layers was demanded. Such a phenomenon explained high water absorption of compomers and high hydroscopic expansion. Ionosit showed the highest values of water sorption among all tested materials. After polymerization the volume of material increased and this process compensated the polymerization contraction of polymer phase. This feature might be explained by the increased water sorption. Dyract eXtra differed from all tested compomers: low water absorption value and slow, consequent reduction in contraction stress only up to 1.6 MPa after 21 days of water immersion. These results could be explained by low filler content in comparison to the other compomers.

The photoelastic method turned out to be inadequate to evaluate stress changes of RMGI (Fuji II LC and Ionolux). Consequently, a different method allowing for observation of the state stress development in RMGI during water aging is highly needed. An alternative measurement technique might be a tensiometer [27,28], strain gauges [29,30] and a stress–strain analyser [31,32].

A major drawback associated with resin dental materials was polymerization shrinkage. It could cause a marginal gap formation. Water sorption might help compensate the hygroscopic expansion [33]. Moreover, it also relieved polymerization shrinkage stress generated along cavity walls during the initial setting stage. However, water absorbed into resin matrices, acts as a plasticizer adversely effecting on the materials’ physical properties [34]. Furthermore, the excessive expansion could create stress that might possibly result in undesirable cuspal flexure and fracture of unsupported tooth structure [23,35].

## 5. Conclusions 

The water sorption, solubility and absorption dynamic of ion-releasing restorative materials were material dependent properties. The overall results indicated that the tested restorative materials showed significant stress decrease. The total reduction in contraction stress or water expansion stress was not observed for materials with low value of water sorption (Beautifil Bulk Fill, Dyract eXtra, Glasionosit and Twinky Star). The photoelastic method turned out to be inadequate to evaluate stress changes of RMGI.

## Figures and Tables

**Table 1:** The composition and curing time of investigated materials.

Material	Manufacturer (Country)	Type	Composition	Curing Time [s]
Beautifil Bulk Fill Flow	Shofu (Japan)	Giomer	bis-GMA, UDMA, bis-MEPEPP, TEGDMA, multi-functional glass filler and S-PRG filler based fluoro-alumino-silicate glass (73 wt %)	10
Beautifil Flow Plus F00	Shofu (Japan)	Giomer	bis-GMA, TEGDMA, multifunctional glass filler, improved S-PGR filler based on aluminofluoro-borosilicate glass, Al_2_O_3_ (67.3 wt %, 47.0 vol %)	10
Beautifil Flow F02	Shofu (Japan)	Giomer	bis-GMA, TEGDMA, multifunctional glass filler, improved S-PGR filler based on fluoro-boroaluminosilicate glass (54.5 wt %/34.6 vol %)	10
Dyract eXtra	Dentsply Sirona (USA)	Compomer	UDMA, carboxylic acid modified, dimethacrylate resin, TEGDMA, BHT, strontium alimino-sodium-fluoro-silicate glass (50 vol %)	10
Compoglass Flow	Ivoclar Vivadent (Germany)	Compomer	UDMA, PEGDMA, cycloaliphate dicarbonic acid dimethacrylate, catalysts, stabilizers and pigments, mixed oxide—silanized, ytterbiumtrifluoride, Ba-Al-fluorosilikateglass-silanized (66.8 wt %)	20
Ionosit	DMG (Germany)	Compomer	acrylic resin, glass powder, silica, aliphatic dimethacrylate, aromatic dimethacrylate, polycarboxylic polymethacrylate (72 wt % 55 vol %)	20
Glasiosite	Voco (Germany)	Compomer	bis-GMA, UDMA, TEGDMA, BHT, SiO_2_, (Ba,B)AlSi, FAlSi (77.5 wt%)	20
TwinkiStar	Voco (Germany)	Compomer	bis-GMA, UDMA, carboxylic acid modified methacrylate, camphorquinone, BHT, Ba-Al- Str-fluorosilicate glass, Silicon dioxide (78 wt %)	20
Ionolux	Voco (Germany)	RMGI	polyacrylic acid, HEMA, bis-GMA, UDMA, fluoro-alumino-silicate glass	20
Fuji II LC	GC (USA)	RMGI	polyacrylic acid, HEMA, UDMA, camphorqunone, fluoro-alumino-silicate glass	20

bis-GMA—bisphenol A glycol dimethacrylate, bis-MPEPP—bisphenol A polyethoxy methacrylate, TEGDMA—triethylene glycol dimethacrylate, UDMA—urethane dimethacrylate, PEGDMA—polyethylene glycol dimethacrylate, BHT—butylated hydroxytoluene, HEMA—hydroxyethylmethacrylate.

**Table 2:** The composition and curing time of bonding systems.

Material	Manufacturer (Country)	Dedicated Restorative Material	Composition	Curing Time [s]
BeautiBond	Shofu (Japan)	Beautifil Flow, Beautifil Bulk Fill Flow, Beautifil Flow Plus F00	bis-GMA, TEGDMA, phosphoric acid monomer, carboxylic acid monomer	10
XP Bond	Dentsply Sirona (USA)	Dyract eXtra, Fuji II LC	TCB, PENTA, UDMA, TEGDMA, HEMA, butylated benzenediol (stabilizer), ethyl-4-dimethylaminobenzoate, camphorquinone	10
Monobond Plus	Ivoclar Vivadent (Germany)	Compoglass Flow	10-MDP, silane methacrylate, ethanol, sulfide methacrylate	10
Ecosite-Bond	DMG (Germany)	Ionosit	dental resins, ethanol, water, additives and catalysts	10
Futurabond M+	Voco (Germany)	Glasiosite, TwinkyStar, Ionolux	HEMA, bis-GMA, etanol, acidic adhesive monomer	10

**Table 3:** Stress state before (0.5 h) and after 2016 h (84 days) of water immersion, contraction stress drop, absorbency and solubility of tested materials.

Material	Stress State [MPa]		Absolute Values of Stress Changes [MPa]	Contraction Stress Drop [%]	Sorption [µg/mm^3^]	Solubility [µg/mm^3^]
	0.5 h	2016 h				
Beautifil Flow F02	16.7 ± 0.9	−4.7 ± 0.8	21.4	128 *	45.9 ± 2.1	0.2 ± 0.1
Beautifil Bulk Fill Flow	11.1 ± 0.4	4.2 ± 0.9	6.9	62	13.6 ± 0.4	0.9 ± 0.3
Beautifil Flow Plus F00	12.8 ± 0.4	0.0 ± 0.2	12.8	100	26.4 ± 1.0	0.5 ± 0.1
Dyract eXtra	7.8 ± 0.1	1.6 ± 0.1	6.2	79	16.5 ± 0.5	2.9 ± 0.7
Compoglass Flow	10.4 ± 0.9	−0.4 ± 0.2	10.8	104 *	28.9 ± 0.8	2.3 ± 0.5
Ionosit	13.4 ± 1.1	−4.7 ± 0.2	18.1	135 *	103.8 ± 0.9	3.0 ± 0.2
Glasiosite	9.5 ± 0.2	−0.5 ± 0.2	10.0	105 *	16.4 ± 0.8	1.3 ± 0.1
TwinkiStar	7.9 ± 0.2	0.0 ± 0.2	7.9	100	17.7 ± 0.4	1.6 ± 0.8

* materials with over-compensated water expansion.
